# Stunting among kindergarten children in China in the context of COVID-19: A cross-sectional study

**DOI:** 10.3389/fped.2022.913722

**Published:** 2022-08-02

**Authors:** Xueyan Ma, Xiangzheng Yang, Hongzhi Yin, Yang Wang, Yuanshuo Tian, Chaojun Long, Chen Bai, Fei Dong, Zhendong Wang, Tiegang Liu, Xiaohong Gu

**Affiliations:** ^1^School of Traditional Chinese Medicine, Beijing University of Chinese Medicine, Beijing, China; ^2^Department of Pediatrics, Beijing University of Chinese Medicine Shenzhen Hospital (Longgang), Shenzhen, China; ^3^Beijing University of Chinese Medicine Third Affiliated Hospital, Beijing University of Chinese Medicine, Beijing, China; ^4^Dongzhimen Hospital, Beijing University of Chinese Medicine, Beijing, China

**Keywords:** stunting, kindergarten children, malnutrition, coronavirus disease 2019, eating behavior, feeding behavior

## Abstract

**Background:**

The impact of COVID-19 has most likely increased the prevalence of stunting. The study aimed to determine the prevalence of stunting among kindergarten children in the context of coronavirus disease 2019 (COVID-19) in Longgang District, Shenzhen, China, and its risk factors.

**Methods:**

A cross-sectional study was conducted to identify children from 11 sub districts of 481 kindergartens in the Longgang District of Shenzhen City from May to July 2021. In the context of COVID-19, an online survey was conducted to gather demographic information, height, birth information, and lifestyle. The prevalence of stunting was calculated, and the risk factors were analyzed using binary logistic regression with three stepwise models.

**Results:**

A total of 118,404 subjects were included from May to July 2021, with a response and questionnaire effective rates of 85.75% and 95.03%, respectively. The prevalence of stunting and severe stunting were 3.3% and 0.8%, respectively. Model 3 showed that risk factors for stunting were male sex [odds ratio (OR) = 1.07], low birth weight (OR = 2.02), insufficient sleep time (OR = 1.08), less food intake than their peers (OR = 1.66), slower eating than their peers (OR = 1.16), accompanied by grandparents alone or non-lineal relatives (reference: parents accompanying) (OR = 1.23, 1.51), and children induced to eat (OR = 1.17). Protective factors included only-child status (OR = 0.66), reported high activity (OR = 0.37, 0.26, 0.23), parents with high education levels (father: OR = 0.87, 0.69; mother: OR = 0.69, 0.58), high monthly income per capita of the family (OR = 0.88, 0.74, 0.68), and allowing children to make food choices (OR = 0.82).

**Conclusion:**

The stunting rate of children in kindergartens in Longgang District is 3.3%, close to the level of developed countries but higher than the average level of developed cities in China. The relatively high stunting rate in children under 3 years old in 2021 may be associated with the influence of COVID-19. Appropriate policies should be formulated for individuals and families with children to help children establish good living habits and reduce stunting.

## Introduction

Stunting refers to the height/length-for-age two standard deviations below the median, according to standard growth charts (1). Studies showed that stunting significantly impacts the life and health of children throughout their lives (including adolescence and adulthood) (2–6). Stunting is a major global health priority and was estimated to affect 22.0% or 149.2 million children under 5 years globally in 2020 (7). The World Health Organization (WHO) proposed a 40% reduction in stunted children as the first of six global nutrition targets by 2025 compared with the baseline data in 2010 (8). Although stunting has declined steadily since 2000, there remain challenges to achieving this goal. Coronavirus disease 2019 (COVID-19) increased the difficulty of achieving the goal. The epidemic has impacted social and economic development. Control of public places, the suspension of kindergartens, and the increase of online courses have affected children’s lives and health (9). The WHO reported that the impact of COVID-19 likely increased the prevalence of stunting (7).

Currently, there is a lack of investigations on the stunting rate among preschool children in areas with rapid economic development against the background of the epidemic affecting social and economic operations. Longgang District is in the northeast of Shenzhen. In recent years, its economy has developed rapidly, ranking first among the top 100 industrial regions in China from 2018 to 2021, according to the China Information and Communication Research Institute (10–13). However, due to the impact of COVID-19 and Sino-American trade friction, the gross domestic product in the Longgang District decreased by 7.6% year on year in 2021 (14). In the present study, we aim to perform a cross-sectional study of the prevalence of stunting in the Longgang District in 2021 and identify the risk factors associated with stunting.

## Materials and methods

### Study design

We conducted a cross-sectional study of enrolled preschoolers from kindergartens of Longgang District, Shenzhen City, Guangdong Province, in 2021. There are 11 sub districts and 481 kindergartens in Longgang District. Sporadic COVID-19 cases were reported in Shenzhen during the survey. The control measures in kindergartens are strict. Because of the epidemic, we conducted an online questionnaire survey in kindergartens. The information included height, demographic data, birth information, and lifestyle. The prevalence of stunting and risk factors among preschoolers in the Longgang District was analyzed.

**FIGURE 1:**
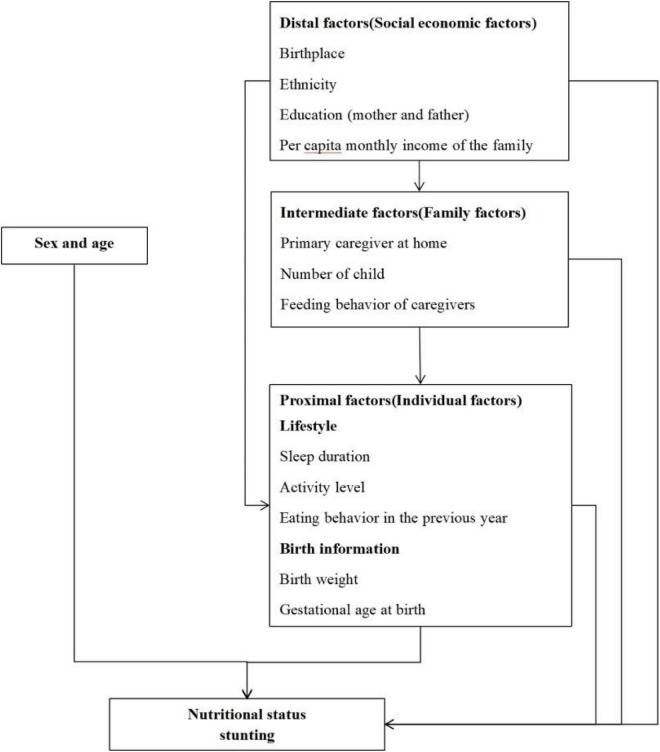
Conceptual framework.

Based on the previous literature (15), this paper constructed a conceptual framework for analyzing factors associated with stunting (Figure 1). The framework provided a way to understand that different factors affect each other and jointly lead to stunting. Distal factors such as birthplace, ethics, parents’ qualifications, and other socioeconomic factors can affect height/length through intermediate and proximal factors. Family factors such as primary caregiver at home, number of children, and feeding behavior of caregivers belong to intermediate factors, which can affect height/length through proximal factors. Proximal factors include children’s lifestyle and birth factors.

### Participants and diagnostic criteria

We investigated children in kindergartens in the Longgang District using a general investigation from May to July 2021. The researcher first contacted the person in charge of all kindergartens in Longgang District, with kindergartens as the unit, and explained the contents and purpose of the survey. If the person in charge of the kindergarten agreed to participate in this survey, all children who met the inclusion and exclusion criteria were included.

The inclusion criteria were as follows: children studying in kindergartens in Longgang District, Shenzhen, in the second semester from 2020 to 2021; guardians possessed reading and writing skills and were willing and able to complete the survey. Exclusion criteria were children who did not complete or submit the questionnaire to the online system.

Criteria for the diagnosis of stunting: children less than 60 months old are judged according to WHO Child Growth Standards (2006 Edition) (16). 5-year-old children (60–71.9 months old) are judged according to WHO Child Growth Standards (2007 Edition). Z-score is the deviation of an individual’s value of the median value of a reference population, divided by the standard deviation of the reference population. If the Z-score of height/length-for-age (HAZ/LAZ) was less than –2, it was considered stunting. If the HAZ was less than –2, it was judged as stunting. For children under 6 years old (less than 72 months old), severe stunting was defined as HAZ less than –3. Children aged 6 and above were judged according to the People’s Republic of China’s “screening for malnutrition in school-age children and adolescents” (WS/T 456-2014). If the child’s height was no more than the cutoff value, the child was judged to be stunted. Stunting was not diagnosed if the child had a clear cause (e.g., genetic metabolic diseases).

### Data collection

All investigators were trained uniformly. Unified standard operating procedures were developed for the investigation process to ensure the quality of the investigation. The investigator introduced the survey to the guardians of the subjects, explained in detail the requirements for filling out the questionnaire, and issued a unique identification code for the subject. The guardians of the subjects can enter the online system to participate in the investigation with the unique identification code. The home page of the electronic survey provided the basic introduction and informed consent form. The subjects who chose to agree to participate filled out the questionnaire.

Data included the following: (1) demographic characteristics (gender, date of birth); (2) height/length to one decimal place in centimeters; (3) distal factors (birthplace, nationality, parents’ qualifications, per capita monthly income of the family); (4) intermediate factors (Primary caregiver at home, Number of children, feeding behavior of caregivers). Feeding behavior of caregivers includes forcing or punishing children to coerce them to eat more, inducing them to eat (e.g., using toys or television), and attitudes toward children’s bad eating habits. These behaviors were recorded if the caregiver engages in such behavior more than 3 days per week. (5) Proximal factors (see below).

(i) Sleep duration
  Adequate sleep for children under 3 years is at least 11 h daily. For children over 3 years of age, more than 10 h a day is sufficient (17, 18).
(ii) Activity level
  There were four activity levels: essential inactivity, less activity, general, and more significant activity.
(iii) Eating behavior in the previous year
  This category includes eating less than their peers, eating slowly, not being interested in food, not eating in fixed places, and inattention while eating. Children demonstrating this behavior more than 3 days a week were considered to have this dietary behavior. The caregivers were asked to report whether there was picky eating.
(iv) Birth information
  Birth information includes birth weight and gestational age at birth. If the birth weight was less than 2.5 kg, it was considered to be low birth weight. It was considered premature if the gestational age at birth was less than 37 weeks.

### Statistical analysis

Categorical data were expressed as percentages and 95% confidence intervals (CI). According to the data type, the chi-square test or Fisher’s exact test was used to compare the differences between groups. Binary logistic regression was used to calculate the correlations between stunting and demographic characteristics, birth status, the lifestyle of children, and the feeding behavior of caregivers in the previous year with three stepwise models based on the conceptual framework. Binary logistic regression was used because the dependent variable, stunting, is dichotomous. Model 1 was constructed with distal factors to estimate their effect on stunting. Model 2 was constructed with distal and intermediate factors. Model 3 was constructed with distal, intermediate, and proximal factors. The statistical analysis was conducted using SPSS 20.0 (IBM, NY, United States). Two-sided *p*-values less than 0.05 were considered to be statistically significant.

## Results

**FIGURE 2:**
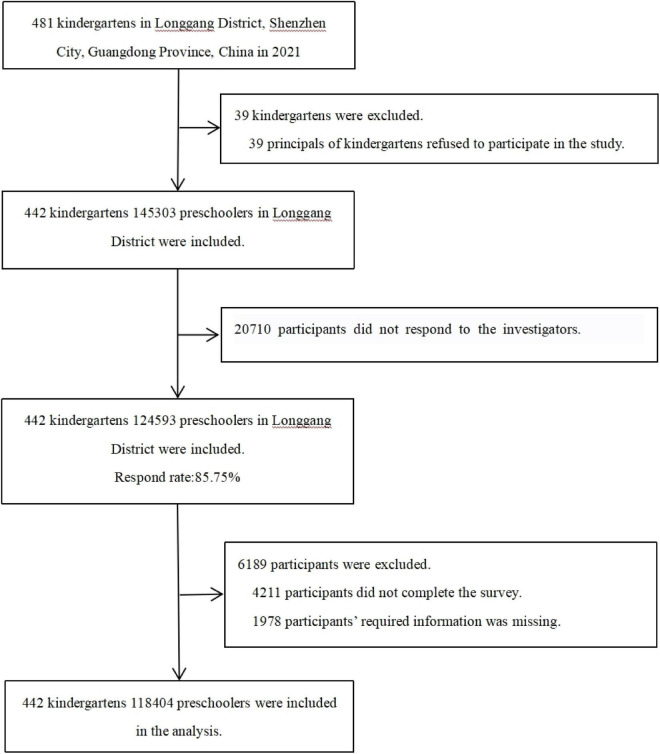
Study population.

A total of 39 (8.11%) of kindergartens in the Longgang District refused to participate in the survey, and 442 (91.89%) agreed to participate. A total of 145,303 children from 442 kindergartens were investigated. A total of 124,593 children participated in the survey, with a response rate of 85.75%. Of these, 4,211 participants failed to complete the survey, and 1,978 subjects lacked critical information such as birth date or height. Finally, 118,404 subjects were included in the study for an effective rate of 95.03% (Figure 2). We set a test question in the questionnaire to reflect the accuracy of the questionnaire. The subjects were required to choose the kindergartens where the children were. A total of 117,870 subjects answered the test item correctly (99.55%).

The prevalence of stunting was 3.3% (95% confidence interval 3.2–3.4%). The rate of stunting in children under 5 years old was 3.6%, and that of children under 6 years old was 3.6%. There were significant differences in stunting rate among ages, education levels of the father and mother, and per capita monthly income of families and primary caregivers of children. There were no significant differences between genders and nationalities (Table 1).

**TABLE 1:** Prevalence of stunting among preschoolers in Longgang District, Shenzhen City, China in 2021 (*n* = 118,404).

Characteristics	No of participants	No with stunting	Prevalence,% (95% CI)	*P*-value
Overall	118,404	3,912	3.3 (3.2–3.4)	
Age (years)				<0.001
Age < 3	158	6	3.8 (0.8–6.8)	
3 ≤ age < 6	88,361	3,228	3.7 (3.5–3.8)	
6 ≤ age	29,885	678	2.3 (2.1–2.4)	
Sex				0.91
Male	63,184	2,084	3.3 (3.2–3.4)	
Female	55,220	1,828	3.3 (3.2–3.5)	
Ethnicity				0.28
Han nationality	112,836	3,714	3.3 (3.2–3.4)	
Other nationalities	5,568	198	3.6 (3.1–4.0)	
Education (father)				<0.001
Junior high school and below	20,744	1,087	5.2 (4.9–5.5)	
High school and junior college	55,864	1,903	3.4 (3.3–3.6)	
Bachelor’s degree or above	41,796	922	2.2 (2.1–2.3)	
Education (mother)				<0.001
Junior high school and below	22,506	1,240	5.5 (5.2–5.8)	
High school and junior college	60,415	1,898	3.1 (3.0–3.3)	
Bachelor’s degree or above	35,483	774	2.2 (2.0–2.3)	
Per capita monthly income (¥)				<0.001
Less than 5,000	25,887	1,204	4.7 (4.4–4.9)	
5,000 ∼10,000 (5,000 included)	38,549	1,358	3.5 (3.3–3.7)	
10,000 ∼ 20,000 (10,000 included)	30,648	768	2.5 (2.3–2.7)	
No less than 20,000	14,121	297	2.1 (1.9–2.3)	
Not filled in	9,199	285	3.1 (2.7–3.5)	
The primary caregiver at home				<0.001
Father/mother	101,705	3,305	3.2 (3.1–3.4)	
Ancestors (Grandpa/Grandma)	12,951	473	3.7 (3.3–4.0)	
Parents and grandparents	2,505	71	2.8 (2.2–3.5)	
Others (non-immediate family, Nanny)	1,243	63	5.1 (3.8–6.3)	

Among 88,519 children under 6 years of age, the prevalence of severe stunting was 0.8% (0.7%–0.9%). There were significant differences in the rate of severe stunting among children under 6 years old in gender, education level of the father and mother, and per capita monthly income of family and primary caregivers of children; however, there was no significant difference among nationalities (Table 2).

**TABLE 2:** Severe stunting rate for children under 6 years of age in Longgang District, Shenzhen City, China in 2021 (*n* = 118,404).

Characteristics	No. of participants	No. with severe stunting	Prevalence,% (95% CI)	*P*-value
Overall	88,519	713	0.8 (0.7–0.9)	
Sex				0.008
Male	46,907	413	0.9 (0.8–1.0)	
Female	41,612	300	0.7 (0.6–0.8)	
Ethnicity				0.14
Han nationality	84,330	671	0.8 (0.7–0.9)	
Other nationalities	4,189	42	1.0 (0.7–1.3)	
Education (father)				<0.001
Junior high school and below	14,382	204	1.4 (1.2–1.6)	
High school and junior college	41,578	349	0.8 (0.8–0.9)	
Bachelor’s degree or above	32,559	160	0.5 (0.4–0.6)	
Education (mother)				<0.001
Junior high school and below	15,614	241	1.5 (1.4–1.7)	
High school and junior college	45,050	350	0.8 (0.7–0.9)	
Bachelor’s degree or above	27,855	122	0.4 (0.4–0.5)	
Per capita monthly income (¥)				<0.001
Less than 5,000	18,734	238	1.3 (1.1–1.4)	
5,000–10,000 (5,000 included)	28,911	233	0.8 (0.7–0.9)	
10,000–20,000 (10,000 included)	23,143	135	0.6 (0.5–0.7)	
No less than 20,000	10,610	54	0.5 (0.4–0.6)	
Not filled in	7,121	53	0.7 (0.5–0.9)	
The primary caregiver at home				<0.001
Father/mother	75,266	586	0.8 (0.7–0.8)	
Ancestors (Grandpa/Grandma)	10,361	103	1.0 (0.8–1.2)	
Parents and grandparents	1,972	10	0.5 (0.2–0.8)	
Others (non-immediate family, Nanny)	920	14	1.5 (0.7–2.3)	

In model 3, binary multivariate logistic regression showed that showed that risk factors for stunting were male sex [odds ratio (OR) = 1.07], low birth weight (OR = 2.02), insufficient sleep time (OR = 1.08), less food intake than their peers (OR = 1.66), slower eating than their peers (OR = 1.16), accompanied by grandparents alone or non-lineal relatives (reference: parents accompanying) (OR = 1.23, 1.51), and children induced to eat (OR = 1.17). Protective factors included only-child status (OR = 0.66), reported high activity (OR = 0.37, 0.26, 0.23), parents with high education levels (father: OR = 0.87, 0.69; mother: OR = 0.69, 0.58), high monthly income per capita of the family (OR = 0.88, 0.74, 0.68), and allowing children to make food choices (OR = 0.82) (Table 3).

**TABLE 3:** Logistic regression models for stunting among preschoolers in Longgang District, Shenzhen City, China, in 2021 (*n* = 118,404).

	Model 1		Model 2		Model 3	
Characteristics	Adjusted odds ratio (95% CI)	*P*-value	Adjusted odds ratio (95% CI)	*P*-value	Adjusted odds ratio (95% CI)	*P*-value
Age (reference: age < 3 years)						
3 years ≤ age < 6 years	1.07 (0.47 to 2.42)	0.88	1.04 (0.46 to 2.36)	0.93	1.00 (0.44 to 2.28)	0.10
6 years ≤ age	0.61 (0.27 to 1.38)	0.23	0.60 (0.26 to 1.36)	0.22	0.57 (0.25 to 1.30)	0.18
Sex (reference: female)						
Male	0.99 (0.93 to 1.06)	0.81	1.00 (0.93 to 1.06)	0.89	1.07 (1.00 to 1.142)	0.04
Ethnicity (reference: Han nationality)						
Other nationalities	1.05 (0.91 to 1.21)	0.52	1.06 (0.92 to 1.23)	0.41	1.07 (0.92 to 1.24)	0.38
Father’s education (reference: junior high school and below)						
High school and junior college	0.85 (0.78 to 0.93)	<0.001	0.86 (0.78 to 0.94)	0.001	0.87 (0.79 to 0.95)	0.002
Bachelor’s degree or above	0.66 (0.59)	<0.001	0.67 (0.60 to 0.76)	<0.001	0.69 (0.61 to 0.77)	<0.001
Mother’s education (reference: junior high school and below)						
High school and junior college	0.68 (0.62 to 0.74)	<0.001	0.69 (0.63 to 0.75)	<0.001	0.69 (0.63 to 0.76)	<0.001
Bachelor’s degree or above	0.56 (0.49 to 0.63)	<0.001	0.57 (0.50 to 0.64)	<0.001	0.58 (0.51 to 0.65)	<0.001
Per capita monthly income of the family (¥) (reference:<5,000)						
5,000–10,000 (5,000 included)	0.84 (0.78 to 0.91)	<0.001	0.86 (0.79 to 0.93)	<0.001	0.88 (0.81 to 0.96)	0.002
10,000–20,000 (10,000 included)	0.68 (0.62 to 0.75)	<0.001	0.70 (0.64 to 0.77)	<0.001	0.74 (0.67 to 0.81)	<0.001
No less than 20,000	0.62 (0.54 to 0.71)	<0.001	0.64 (0.56 to 0.73)	<0.001	0.68 (0.59 to 0.78)	<0.001
Not filled in	0.81 (0.71 to 0.93)	0.002	0.85 (0.74 to 0.97)	0.01	0.87 (0.75 to 0.10)	0.05
The primary caregiver at home (reference: parents)						
Ancestors (Grandpa/Grandma)	NA	NA	1.25 (1.13 to 1.39)	<0.001	1.23 (1.12 to 1.37)	<0.001
Parents and grandparents	NA	NA	0.89 (0.70 to 1.13)	0.33	0.90 (0.71 to 1.14)	0.37
Others	NA	NA	1.52 (1.18 to 1.97)	0.001	1.51 (1.17 to 1.96)	0.002
Only child	NA	NA	0.67 (0.61 to 0.73)	<0.001	0.66 (0.61 to 0.72)	<0.001
Birthplace (reference: Shenzhen)						
Other cities in China	1.01 (0.94 to 1.08)	0.88	1.04 (0.97 to 1.11)	0.28	1.04(0.97 to 1.12)	0.22
Other countries	0.87 (0.32 to 2.36)	0.79	0.85 (0.32 to 2.31)	0.75	0.84 (0.31 to 2.27)	0.73
Fetal age (reference: full-term)						
Premature birth (<37 weeks)	NA	NA	NA	NA	0.87 (0.76 to 1.00)	0.05
Birth weight (reference: ≥ 2.5 kg)						
Low birth weight (<2.5 kg)	NA	NA	NA	NA	2.02 (1.82 to 2.24)	<0.001
Physical activities (reference: Basically inactive)						
Less activity	NA	NA	NA	NA	0.37 (0.18 to 0.73)	0.005
General activity	NA	NA	NA	NA	0.26 (0.13 to 0.51)	<0.001
More intense activity	NA	NA	NA	NA	0.23 (0.11 to 0.45)	<0.001
Not filled in	NA	NA	NA	NA	0.26 (0.13 to 0.54)	<0.001
Sleep time (reference: sufficient)						
Not enough	NA	NA	NA	NA	1.08 (1.01 to 1.15)	0.03
Not filled in	NA	NA	NA	NA	2.87 (0.99 to 8.34)	0.05
Children eating behavior						
Supplementation of nutrients alone	NA	NA	NA	NA	1.00 (0.93 to 1.08)	0.99
Eating less than their peers	NA	NA	NA	NA	1.66 (1.50 to 1.84)	<0.001
Eating slower than their peers	NA	NA	NA	NA	1.16 (1.06 to 1.27)	0.001
Not interested in food	NA	NA	NA	NA	1.07 (0.96 to 1.20)	0.22
Distraction when eating (watch TV, play games)	NA	NA	NA	NA	1.02 (0.93 to 1.11)	0.71
An irregular dining place	NA	NA	NA	NA	1.10 (0.97 to 1.25)	0.13
Caregiver’s judgment on whether the child is picky about food (reference: no)						
Picky food	NA	NA	NA	NA	1.01 (0.93 to 1.09)	0.78
Not sure	NA	NA	NA	NA	1.12 (0.95 to 1.31)	0.19
Feeding behavior of caregivers						
Little emotional exchange during the meal	NA	NA	1.08 (0.97 to 1.20)	0.18	1.02 (0.91 to 1.14)	0.78
Forcing or punishing children to eat	NA	NA	1.35 (1.17 to 1.56)	<0.001	1.02 (0.88 to 1.18)	0.79
Inducing children to eat (toys, television, story reward)	NA	NA	1.39 (1.24 to 1.57)	<0.001	1.17 (1.04 to 1.33)	0.01
Allowing children to choose their food at will	NA	NA	0.88 (0.80 to 0.97)	0.01	0.82 (0.75 to 0.90)	<0.001
Allowing children to snack freely	NA	NA	1.01 (0.86 to 1.18)	0.92	0.99 (0.85 to 1.16)	0.92
Allowing children to hang out while eating	NA	NA	1.06 (0.87 to 1.29)	0.55	0.95 (0.78 to 1.16)	0.62

## Discussion

### Prevalence of stunting

Our main finding was that the incidence of stunting among kindergarten children in the Longgang District was 3.3% (3.2–3.4%). The stunting rate of children under 5 years old was 3.6%, significantly lower than that in the world in 2020 (22%) and lower than in East Asia in 2020 (4.9%). The incidence was close to the level of North America (3.2%) and slightly higher than those in northern Europe, Australia, and New Zealand (2.3%–2.9%) (7). Overall, the stunting rate of kindergarten children in Longgang District was significantly lower than that in most developing countries and close to or slightly higher than that in developed countries. The relatively low stunting rate in the Longgang District of China may be associated with improved living standards by improving China’s economic level and children’s health care services. In recent years, China’s economic level has developed rapidly, and Shenzhen is in a golden development period of the superposition of Shenzhen Special Economic Zone and Shenzhen Pioneer Demonstration Zone. Economic development improves living standards, food supply, and quality. The Chinese government launched a series of nutrition improvement projects for Chinese children in several regions, providing good conditions for children’s growth and development.

The stunting rate of children under 6 years old in Longgang District was 3.6%, and that of children over 6 years old was 2.3%. The stunting rate of children under 6 years old was lower than that of children under 6 years old in China reported in 2020 (4.8% Note: The data come from the monitoring of nutrition and health status of Chinese residents from 2015 to 2017), and is comparable to that of urban prevalence (3.5%) (19). The stunting rate of children in Longgang District is higher than that in developed regions of China at 0.4% in 2016 and is comparable to the rate in Hunan Province of China of 3.1% in 2019 (20, 21).

The prevalence of stunting is strongly inversely correlated with the region’s wealth (22). As a region with a developed economy in China, the stunting rate of preschool children in the Longgang District is higher than the average level of developed areas in China, which might be related to the demographic characteristics of the Longgang District. The population mobility of Longgang District is relatively large, and its permanent non-registered population is as high as 2,897,300, accounting for 72.45% of the permanent population, which ranks high nationwide (23). The floating population faces several obstacles to obtaining comprehensive, affordable public health services, and this is one of the weakest links in China’s public health service system (23, 24). Studies showed that immigrant children’s growth and health level might be affected by the relatively unstable living environment and the shortage of medical and health services (25–28).

The COVID-19 epidemic has little effect on the overall stunting rate in Longgang District. A nutritional survey on kindergarten children in Shenzhen was carried out in 2015 (29). A stratified random sampling method was used. The kindergartens were divided into 11 layers according to their affiliated maternal and child health hospital, and 1/4 of kindergartens were randomly assigned in each layer. Kindergartens in the Longgang District were one of the layers. Seventy-nine kindergartens were assigned randomly from 318 kindergartens in the Longgang District in the survey. The height was measured by the maternal and child healthcare system staff, and the measurement method was standardized. Criteria for the diagnosis of stunting were according to the WHO Child Growth Standards. The diagnostic criteria for children under 6 years were the same as in this study; The diagnostic criteria for children aged six and above were similar. So that the data in 2015 and 2021 are comparable. The prevalence of stunting among kindergarten children in the Longgang District of Shenzhen in 2021 was 3.3%, lower than that in 2015 (4.71%) (29). The relatively low stunting rate in 2021 may be associated with improved kindergarten construction in Shenzhen City. In recent years, the Education Bureau of Shenzhen Municipality has printed and distributed documents like *The Action Plan for the Development of Preschool Education in Shenzhen (2019–2020)* to vigorously develop the kindergartens’ construction. In 2015, private kindergartens accounted for more than 90% in Shenzhen, while this survey shows that public kindergartens have reached nearly 50%. The government has strengthened the health care work and dietary management in kindergartens, providing strong support for improving children’s nutritional status.

Compared with the data of Shenzhen in 2015, the stunting rate of children under 3 years old in Longgang District increased nearly fourfold (3.8% vs. 1.0%). The relatively high stunting rate in 2021 may be associated with the influence of COVID-19. Social quarantine measures during the epidemic have significantly impacted children’s living conditions and lifestyles. Studies showed that during COVID-19, children’s physical activity (PA) decreased significantly, while the time spent watching electronic screens increased and unhealthy diets increased (30). We found that the COVID-19 epidemic significantly impacted the stunting rate of children under 3 years old in kindergartens, which suggests that more attention should be paid to the growth and development of children under 3 years old during the epidemic period.

### Distal factors

The high education level of parents and per capita monthly income are related to stunting. The high education level of the father and mother was a protective factor, consistent with the results of observational studies (31, 32). This finding may be due to the higher education level of parents, who may acquire more nutrition knowledge conducive to scientific feeding. Higher per capita monthly income was a protective factor for stunting, similar to previous studies (33, 34). The higher the family’s monthly income, the more parents can spend on nutrition and better feeding environments.

### Intermediate factors

Primary caregivers at home and the number of children are associated with stunting. Compared with care by parents alone, care by grandparents alone or non-immediate relatives was a risk factor for stunting. Grandparental care, which has become a worldwide social phenomenon, gives rise to poor dietary behaviors and lower levels of physical activity (35, 36). Only-child status was a protective factor for stunting, similar to previous studies (37).

The feeding behavior of caregivers in the previous year is associated with stunting. Encouraging children to eat using incentives such as toys and television is a risk factor, while allowing children to choose food at will is a protective factor. The feeding pattern of parents’ long-term reward inducing children to eat will reduce children’s enjoyment of food, which is not conducive to establishing long-term good eating behavior. The allowable feeding style provides children with a more relaxed eating environment and improves their food enjoyment (38). However, it may lead to a reduction in the diversity of food intake. More studies are needed to explore the association between stunting and allowable feeding style.

### Proximal factors

The present study shows that the risk of boys suffering from childhood stunting is 1.07 times that of girls, the same as many results from China (20, 21, 39, 40) and other regions (41, 42), probably due to the interaction of biological and socio-cultural factors. We also found differences between boys and girls in growth trajectories and immune function beginning prenatally (43, 44). Caregivers treat children of different genders differently (45–47).

Low birth weight is a risk factor for stunting in children, consistent with many other studies (39, 40, 48). Birth weight is an essential indicator of fetal intrauterine nutrition (49). Low birth weight children always have poor digestion ability and low immunity, which may affect their growth and development (50, 51).

We found that lifestyle factors such as insufficient sleep, less PA, less food intake, and slower eating than peers were risk factors for stunting. There are few studies on the correlation between height and sleep deprivation (52). A cohort study of children found no correlation between sleep deprivation and height (53), different from the results of the present study; more studies are needed to explore the relationship between them. PA is associated with children’s growth and development. Studies showed that less active children are shorter, and PA-related epiphyseal loading positively affects the growth of healthy children (54). Poor dietary habits such as eating less and slowly can reduce the diversity of food intake and affect children’s growth and development. Studies showed that measures such as closing kindergartens in the early stage of the epidemic of COVID-19 impacted children’s activities, sleep, and other aspects; short-term changes in PA and sedentary behavior may become permanently involved (55, 56).

This study has some limitations. First, the study was a web-based questionnaire. Compared to the paper version of the questionnaire, this method is more likely to attract responses from younger and more educated people (57, 58); this approach may underestimate the incidence of stunting. Second, a web-based survey cannot rule out the possibility of parents misreporting the children’s heights. However, some major clinical characteristics reported by the participants were in line with the current survey in China. In addition, the correct rate of the questionnaire test was 99.55%, reflecting the accuracy of the survey results. Third, the study focused on the correlation between children’s lifestyle and family environment and stunting; other relevant factors should be included in the future. Finally, although the results gave some insights into the effect of COVID-19 on stunting in kindergarten children, we did not measure the influence of the COVID-19 pandemic in this study.

The stunting rate of children in kindergartens in Longgang in 2021 was low, close to or slightly higher than that of developed countries in the world, but higher than that of other developed cities in China. This finding may be due to the high proportion of the floating population. The stunting rate of children under 3 years old in kindergarten increased significantly, possibly due to the epidemic of COVID-19. Stunting is associated with distal, proximal, and intermediate factors. From a policy perspective, our findings suggest that public health services for migrant children and children under 3 years old should be promoted during the epidemic. Health education for parents with low education levels should be strengthened, and pro-poor policies should be formulated to reduce the effect of distal factors like parents’ education and family income. We should strengthen the guidance of caregivers’ feeding behavior. In addition to parents, grandparents, and other caregivers should be targets of feeding behavior guidance to reduce the effect of intermediate factors. To reduce the effect of proximal factors, we should help children cultivate a healthy lifestyle, including sleep, activity, and eating behavior, through kindergarten and family education. Health management services for boys and maternal health care services should be strengthened to prevent stunting.

## Data availability statement

The raw data supporting the conclusions of this article will be made available by the authors, without undue reservation.

## Ethics statement

The Ethics Review Committee of Shenzhen Hospital of Beijing University of Traditional Chinese Medicine (Longgang) approved the study (SZLDH2021LSYM-030). Written informed consent from the participants or their legal guardian/next of kin was not required to participate in this study in accordance with the national legislation and the institutional requirements.

## Authors contributions

TL and XG conceptualized and designed the study andreviewed the manuscript. XM and XY drafted the initial manuscript, coordinated and supervised data collection, and analyzed the data. HY, YW, YT, CL, CB, FD, and ZW participated in data collection and analysis. All authors read and approved the final manuscript.

## Abbreviations

COVID-19: coronavirus disease 2019
WHO: World Health Organization
HAZ: height-for-age
LAZ: length-for-age
CI: confidence intervals
OR: odds ratio
PA: physical activity.
